# ZnO Metal Oxide Semiconductor in Surface Acoustic Wave Sensors: A Review

**DOI:** 10.3390/s20185118

**Published:** 2020-09-08

**Authors:** Izabela Constantinoiu, Cristian Viespe

**Affiliations:** National Institute for Laser, Plasma and Radiation Physics, Laser Department, Atomistilor 409, 077125 Magurele, Romania; izabela.constantinoiu@inflpr.ro

**Keywords:** surface acoustic wave, gas, sensor, metal oxide semiconductor, ZnO

## Abstract

Surface acoustic wave (SAW) gas sensors are of continuous development interest to researchers due to their sensitivity, short detection time, and reliability. Among the most used materials to achieve the sensitive film of SAW sensors are metal oxide semiconductors, which are highlighted by thermal and chemical stability, by the presence on their surface of free electrons and also by the possibility of being used in different morphologies. For different types of gases, certain metal oxide semiconductors are used, and ZnO is an important representative for this category of materials in the field of sensors. Having a great potential for the development of SAW sensors, the discussion related to the development of the sensitivity of metal oxide semiconductors, especially ZnO, by the synthesis method or by obtaining new materials, is suitable and necessary to have an overview of the latest results in this domain.

## 1. Introduction

The increased level of pollution, the development of military resources, and the detection of certain diseases are just some of the areas in which sensors with remarkable sensitivities are being developed. Prevention is the best measure against calamities or any imminent dangers for society and individuals. Consequently, the development of sensors with the highest possible sensitivities and selectivities depending on the applications and with the lowest possible response time, as well as with reversibility, is necessary.

Thus far, sensors have been developed for several domains, such domestic safety, societal security, industry, and environmental control [1,2,3,4,5]. Depending on the measurement data, gas sensors are classified into resistive sensors [6,7], surface acoustic wave (SAW) sensors [8,9], chemiresistive sensors [10,11], electrochemical sensors [12,13], calorimetric sensors [14,15], thermal conductivity sensors [16,17], optical sensors [8,18], etc. Since 1979, when the SAW technology was applied in sensing applications by Wohltjen and Dessy [19,20], there has been growing interest in obtaining this high-performance type of sensors. A considerable advantage of this type of sensor is that it is versatile, which allows for adaptation to different applications [21]. SAW sensors are used for the detection of gases [22,23,24], as well as for the detection of liquids or other changes in the environment, such as pressure [25], humidity [26], the presence of UV radiation [27], and for chemical and biological applications [28,29]. In this work, we focused on the study of SAW sensors for gas detection.

The performance criteria of SAW sensors are the sensitivity, selectivity, limit of detection (LOD), response time, desorption time, and reversibility [30,31].

In the general structure of a SAW sensor, there is a sensitive element where changes occur in the presence of the analyte. This is one of the most important elements of the sensor, which requires development to ensure performance. Over time, different materials have been used for the sensitive element: semiconductor metal oxides, metals, polymers, composite materials, etc. [31,32,33]. The most widely used materials in this field are metal oxide semiconductors [25]. Their advantages are due to the presence of free electrons on their surface, which interact with reducing or oxidative gas molecules [34]. In addition to the nature of the material, of great importance is the morphology of the deposited layer.

A typical process for gas detection takes place by the diffusion of gases from the environment to the sensitive surface, followed by their adsorption and the reaction of the active oxygen species on the surface of the film with gas molecules, thus leading to the modification of the properties of the sensitive material [1,34]. Consequently, a material used in the field of sensors must have pores or voids that facilitate the diffusion of gas molecules, a large specific surface area, and many active areas that lead to the improvement of the interaction between material and gas [1,34,35].

In this paper, we will present, based on recently published results, the development of SAW sensors, specifically those based on ZnO sensitive layers, which is one of the materials often used in the field of sensors. Taking into account the highlighted results, the perspectives for further study will also be described.

## 2. SAW Sensors for Gas Detection

SAW sensors are studied due to their important advantages, such as high sensitivity, short response and recovery times, small size, low cost, and possibility of wireless operation, as well as adaptation to modern manufacturing methods [31,36,37]. In addition, by choosing the substrate, the sensitive layer and the optimal interdigital transducers (IDTs), an improvement of selectivity, response, reversibility, stability, etc. can be obtained [31].

The SAW sensors mainly consist of a piezoelectric substrate, interdigital transducers, and a sensitive surface [30,31].

Piezoelectricity is an electrical polarization that occurs when certain materials are subjected to mechanical stress, and, conversely the onset of deformation in such materials when placed in an electrical field [38,39]. Examples of piezoelectric materials include quartz, LiNbO_3_, LiTaO_3_, La_3_Ga_5_SiO_14_, AlN, GaAs, ZnO [31,40,41,42], etc. The advantages of piezoelectric SAW transducers in principle are the ultra-high sensitivity, proper size and structure, fast response, and compatibility with other technologies and these properties depend on the sensitive layer [33,43].

**Figure 1:**
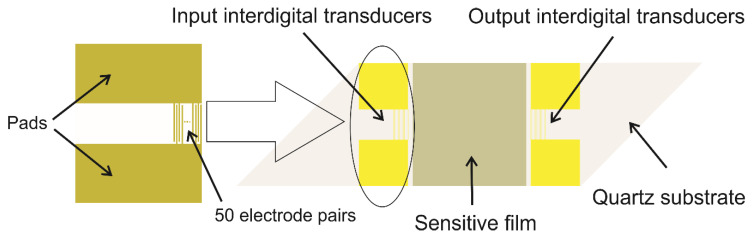
Scheme of a surface acoustic wave (SAW) delay line [44].

A typical “delay line” SAW sensor, as can be seen in Figure 1 [44], consists of an input IDT and an output IDT, both deposited on the piezoelectric substrate of the sensor [30,45]. The surface between the two IDTs is the sensitive surface, onto which a layer is deposited, for the interaction with the analyte. The interaction with the analyte on this area leads to the formation of a delay in time between the input and output signals, which depends on the length of the sensitive layer and the velocity of the SAW [30,31].

IDTs are periodic metallic electrodes deposited on the substrate in the form of two combs intercrossing from opposite sides. Their function is to convert the electrical signal to surface acoustic waves on the piezoelectric surface and then back to an electrical signal [31,46].

The phase velocity and the amplitude of the waves are modified in the presence of an entity at the level of the propagation zone of the acoustic waves. Thus, by recording these changes in the electrical signal at the output IDT, quantitative information regarding the analyte can be obtained [20,30,31,36,47,48]. The frequency shift is directly proportional with the modification of the SAW velocity [30,49]:

(1) $$\frac{\Delta f}{f_{0}}=k\frac{\Delta \nu }{\nu_{0}}$$

where *k* is the fraction of the active surface area used, and

(2) $$\frac{\Delta \nu }{\nu_{0}}=\left(k_{1}+k_{2}\right)f_{0}^{2}h\rho -k_{2}f_{0}^{2}h\left(\left(\frac{4µ}{\nu_{0}^{2}}\right)\;\left(\frac{\lambda +µ}{\lambda +2µ}\right)\right)-k_{2}\frac{\sigma_{s}^{2}}{\sigma_{s}^{2}+\nu_{0}^{2}C_{0}^{2}}$$

where *ν_0_* is the Rayleigh wave velocity in the piezoelectric substrate, *k_1_* and *k_2_* are constants of the piezoelectric substrate, *f_0_* is the unperturbed resonant frequency of the SAW oscillator, *hρ* is the mass per unit area on the surface (where *ρ* is the coating density and h is the coating thickness), *µ* is the shear modulus, *λ* is the first Lame constant of the coating, *σ* is the sheet conductivity of the film, and *C_0_* is the surface capacitance of the substrate.

The efficient combination of the sensitive film with the properties of the sensor substrate are of great importance to obtain good sensor performance. In addition to the sensitivity and selectivity properties, it is necessary for the sensitive layer to fulfil certain physical properties for the sensor to work in the best possible parameters. Thus, the sensitive layer must be thin, uniform, chemically and physically stable when in contact with the test environment, adherent to the substrate, and not cause short-circuiting of the IDTs [30]. Given that the wave propagation front is quite linear, when the layer is not uniform and covers some areas of the wave path to a greater extent than others, this causes delays. This has consequences in terms of the signal-to-noise ratio, which will influence the sensor performance [30]. Research established that the optimum thickness of the sensitive film is about 1% of the wavelength of the wave that travels through the sensor, which depends on the substrate material [30].

The response mechanism of SAW sensors results from the disturbance of the propagation characteristics of the acoustic waves, specifically of wave velocity and attenuation, which result from the interactions at the level of the sensitive film [30,50,51]. The propagation of SAW in a piezoelectric environment can generate both mechanical deformations and electrical potential [30,36]. The interactions that result in mechanical deformations are mass loading and elastic and viscoelastic effects [25,30,52]. The effects resulting from the interaction between the electrical field associated with the SAW and the analyte present at the level of the sensitive film, are defined as acoustoelectric effects [30].

Considering these properties and principles that lead to a correct, high-performance operation of SAW sensors, the development of the sensitive layer from a compositional and morphological point of view will lead to the improvement of the sensitivity and selectivity for different types of gases.

## 3. Sensitive Materials Used in Gas Detection

The development of new materials, with complex and specific properties for a certain application has become a challenge for researchers. For example, the development of materials as thin films on different substrates led to a revolution in several fields, such as catalysts [53], optical layers [54], conductive layers [55], biomedical applications [56], sensors [22], and protective layers [57]. There are many methods that can be used to deposit thin films, including spin coating [58,59], pulsed laser deposition [60,61], dip-coating [62,63], chemical vapor deposition [64,65], evaporation [66,67], and Radio Frequency (RF) magnetron sputtering [68,69].

To date, both organic and inorganic materials have been used in the fabrication of sensors. The category of inorganic materials includes semiconductor metal oxides, metals, oxide compounds, and composites [70,71,72]. In the other category, polymers are the most used organic materials in the field of sensors [73]. The biggest disadvantage of sensors based on inorganic materials, especially semiconductor metal oxides, is the high working temperature, which limits their use for different applications where portability is needed [72,73,74].

There are also polymers with semiconductor properties, such as polyaniline (PAni), which can greatly improve the performance of the sensors, having the effect of lowering the working temperature and improving the sensitivity of the device [73]. The sensitivity of the polymers is due to their ability to transport charged carriers along the polymer chain; a considerable advantage is that they are easy to process in the form of thin films [31,73]. On the other hand, polymers also have disadvantages, such as poor chemical stability and mechanical properties, as well as reduced possibilities of processing in different ways [73,75]. The use of organic and inorganic materials is a good option to improve sensor performance. There are already studies reported on inorganic–organic composite materials, with very good results in the fields of optics and electronics [73,76,77,78,79], and also in the field of sensors, even SAW sensors [44,80,81].

For SAW sensors, one of the most important roles in its operation is that of the sensitive layer, which, in most cases, is in the form of a thin film. The most important features of a SAW sensor, sensitivity and selectivity, are affected and improved by means of the sensitive thin film. The development is, therefore, very important in research aimed at obtaining an outstanding performance in the detection of various gases [22,80,82,83]. Although a wide range of materials has been studied related to the field of sensors, this is still an active domain of research, so that a review bringing together recent results in the domain, including the considerable disadvantages still present, is an important starting point for future research. We therefore address, in this paper, the development of ZnO-based materials in SAW sensors and the perspectives they offer to achieve outstanding sensitivities and selectivity.

An important category of materials for the field of sensors is metal oxide semiconductors. These are versatile materials, used in many fields: domestic electronics, medicine, construction materials, sensors for detection of toxic, flammable and explosive gases [14,24,84,85], etc.

Around 1950, the change of conductivity of certain semiconductor materials, such as Ge, when the adsorption and desorption of gases took place on their surface was demonstrated by Brattain and Berdeen [25,86,87]. Later, in 1962, Seiyama at al. [88] demonstrated the ability of ZnO nanomaterials to detect certain gases at 485 °C, with a metal oxide semiconductor gas sensor, obtaining, for propane, better results than by the method commonly used at that time, thermal conductivity. From this point began the deeper study on metal oxide semiconductors in gas sensing, for which remarkable sensitivities were obtained. However, these notable properties were obtained at high temperatures, as interactions at the material–gas interface were too weak at room temperature (RT) [51,86,89,90].

Advantages, such as the small size of particles, low cost, ease of maintenance, chemical and thermal stability, adaptability to microelectronic processing, and online utilization, led to further studies on the improvement of metal oxide semiconductor materials [1,34,86,91]. In most cases, in order to obtain results in sensors based on metal oxide semiconductors, an energy input is needed, which can come from thermal energy or UV excitation. These methods involve higher energy consumption, which affects the cost of the product, as well as the inability to use these materials for certain applications [86].

Operation at RT is the biggest challenge for sensors based on metal oxide semiconductors, and this had led to the development of ways to overcome this inconvenience. These approaches include doping [92,93,94], obtaining heterojunctions from p and n-type semiconductors [95,96], developing special microstructures [97,98], and inducing oxygen vacancies or the use of composite materials [86,99,100]. Among the most used and known methods of synthesis of doped materials are chemical vapor deposition, the hydrothermal method, the sol-gel method, and thermal annealing [101].

The currently used solution is to adjust the structure of the metal oxide through nanotechnology and composite materials [102]. The review of Li et al. [34] presented the design method and mechanism of detecting harmful gases by different nanostructured metal oxides and composites at RT [34]. Metal oxide semiconductor-based composites combine two or more oxides to improve the gas-sensitivity properties. Thus, composites of n-type oxides (In_2_O_3_/SnO_2_, ZnO/SnO_2_, etc.), n-type and p-type oxides (NiO/ZnO, In_2_O_3_/CuO, NiO/WO_3_ etc.), and p-type oxides (Cu_2_O/Co_3_O_4_, NiO/CuO) were investigated. These materials are characterized by a series of oxygen vacancies, both at the surface and at interfaces, which ensures many active places useful in gas detection [34]. Other advantages result from the appearance of defects at the interfaces of oxide nanoparticles, the formation of heterojunctions that accelerate the transfer of electrons between particles, thus, leading to a much faster response to the sensor [34]. In addition, metal oxides are characterized by the formation of pores, due to the agglomeration of nanoparticles, which favors the adsorption and desorption of gas molecules [34].

The combination of metal oxide semiconductor nanoparticles with polymeric layers was also tested for achieving RT vapour sensing by Sadek et al. [103].

In the research of Aaryashree et al. [104] ZnO functionalized oligophenylenevinylene (OPV) demonstrated a poor response to ammonia (NH_3_) at RT. On the other hand, a Zn-OPV composite, which was formed by the functionalized OPV interacting with inorganic ZnO, showed a larger detection range and stronger sensitivity to NH_3_ than OPV or ZnO at RT, due to the cumulation of the sensitive properties of the two materials used. Rg is the resistance in the presence of the target gas, and Ra is the resistance in the presence of the target gas.

Improving the sensitivity of semiconductor metal oxides in gas detection can also be achieved by UV activation. Metal oxide semiconductors absorb UV radiation and produce photo-generated electrons and holes (Equations (3) and (4)) [105]. These electrons will promote the adsorption of oxygen molecules from the air, thus, resulting in improved material sensitivity (Equations (5)) [105]. Yong Zhou et al. [11] prepared a ZnO nanowire-network sensor via airbrush technology to detect trace NO_2_ gas with dry air (N_2_: 78%; O_2_: 21%) or dry N_2_ as the reference gas, both under UV illumination of a chemiresistive gas sensor at RT (25 °C). Compared with the case of air, the sensor presented a much stronger response and larger sensitivity for N_2_, which was mainly ascribed to a smaller baseline resistance and more photogenerated electrons involved in the reaction with adsorbed NO_2_ molecules. In addition, excellent long-term stability and selectivity were displayed in the N_2_ case.

(3) $$O_{2}\left(g\right)+\;e^{-}\;\to O_{2}^{-}\;\left(ad\right)$$

(4) $$h\nu \;\to \;e^{-}+\;h^{+}$$

(5) $$h^{+}+\;O_{2}^{-}\;\left(ad\right)\to O_{2}\;\left(g\right).$$

Selectivity is another parameter that limits the use of metal oxide semiconductors in gas detection. Special attention is required to determine the selectivity of a metal oxide for a certain gas, because they have remarkable sensitivities for a large category of analytes. There are two main methods to obtain selective sensors based on oxide metal semiconductors [14,106]. The first is to obtain materials that are specifically selective for a particular type of gas and that are not sensitive to gases that may be in the atmosphere in which the testing is performed. For this, several parameters are optimized during the synthesis, such as the concentration of doping with certain elements, temperature, development of multilayers, etc. Another method is to develop electronic noses, i.e., the realization of a matrix of different sensors that have different responses to different analytes [14,45,106].

For all types of sensors, including SAW sensors, it is important to operate at RT, and at the same time, to ensure that a signal is obtained at the lowest analyte concentrations. The reference metal oxide semiconductors used in the domain of sensors include In_2_O_3_ [34,107,108,109,110], ZnO [45,111,112,113], Co_3_O_4_ [114,115,116], SnO_2_ [99,117], CuO/Cu_2_O [100,118], TiO_2_ [24,119], NiO [58,120], Fe_2_O_3_ [121,122], and WO_3_ [23,123]. They were most often used to detect volatile organic compounds, hydrogen, ammonia, etc. [25,99].

In the literature, there are studies that present different methods through which gas detection with these materials was obtained at low temperatures, or even at RT [11,83,100,124], also for SAW sensors. For example, Wang et al. [81] developed a SAW sensor based on copper-ion-doped polyaniline/tungsten oxide nanocomposite for NO detection at RT [90]. A SAW sensor with Pd and a WO_3_ multilayer film was developed for hydrogen detection at RT by Miu et al. [123]. Metal oxide semiconductors doped with metal ions (Au, Pd, Pb, etc.) led to the formation of active defects that favored the adsorption of oxygen molecules from the air [34]. Jakubik et al. [125] used bilayers of WO_3_ and Pd films for hydrogen gas sensing with a SAW sensor. The purpose of combining two layers is to shift the working point to a high sensitivity region based on the acousto-electric interactions. Thus, a small change in conductivity could perturb the SAW velocity and lead to larger frequency shifts.

## 4. ZnO in SAW Sensors

ZnO is an n-type II-IV, wide and direct band gap semiconductor of about 3.37 eV with a large excitation binding energy (60 meV) and with high electron mobility; it is one of the most promising materials in the field of sensors and optoelectronics [1,6,73,76,77,78,79,84]. In addition to the advantages listed above, ZnO is also characterized by a relatively simple synthesis process that allows the control of morphology and leads to oxygen vacancies, which are advantages in the field of sensors for gas molecule adsorption [126]. As for the majority of metal oxide semiconductors, it is a challenge to achieve results at RT and good selectivity in the case of ZnO [127]. As can be seen in Table 1, remarkable results have been obtained for several types of sensors, but most operating conditions involved the use of external energy, such as temperature or UV radiation, to increase the sensitivity of the material [128]. As can be seen, both the use of ZnO and SAW sensors together have a very high potential to obtain ppb results.

**Figure 2:**
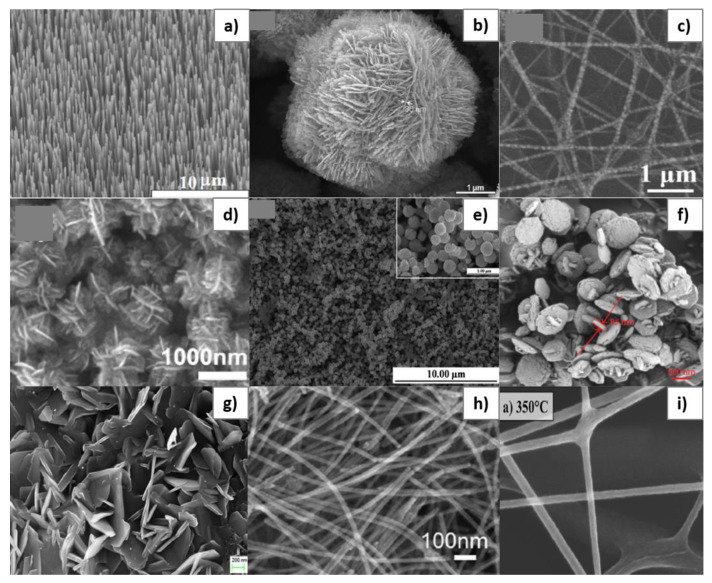
**Scanning Electron Microscope** (SEM) images for different types of morphologies obtained for ZnO: nanorods (**a**) [122], nanosheets (**b**) [132], nanotubes (**c**) [133], nanoflowers (**d**) [1], microspheres (**e**) [134], nanoplates (**f**) [113], nanoflakes (**g**) [135], nanowires (**h**) [136], and nanofibers (**i**) [137]. Reproduced (2020) with permission from Elsevier and ACS Publications.

ZnO has also attracted attention for its low cost, ease of preparation, very good chemical stability, suitability to doping, non-toxicity, and ease of processing, for example in the form of thin films [84,128]. This is a material that can be easily synthesized in various types of nanostructures, such as nanorods (Figure 2a) [122], nanosheets (Figure 2b) [132], nanotubes (Figure 2c) [133], nanoflowers (Figure 2d) [1], microspheres (Figure 2e) [134], nanoplates (Figure 2f) [113], nanoflakes (Figure 2g) [135], nanowires (Figure 2h) [136], and nanofibers (Figure 2i) [137]. These types of microstructures ensure a large specific surface area and allow the penetration of gas molecules into the volume of the material, which is a great advantage for RT detection with SAW sensors [128,138,139,140,141].

To achieve the best sensitivity and RT detection, another method to improve ZnO sensor properties is through the formation of heterostructures and functionalization with metals, such as Au, Pd, or Pt [128,142,143]. In papers that reported improved ZnO sensitivity through such nanostructures, Pd was most often reported as improving the ZnO activity in gas detection [45,119,122].

**Figure 3:**
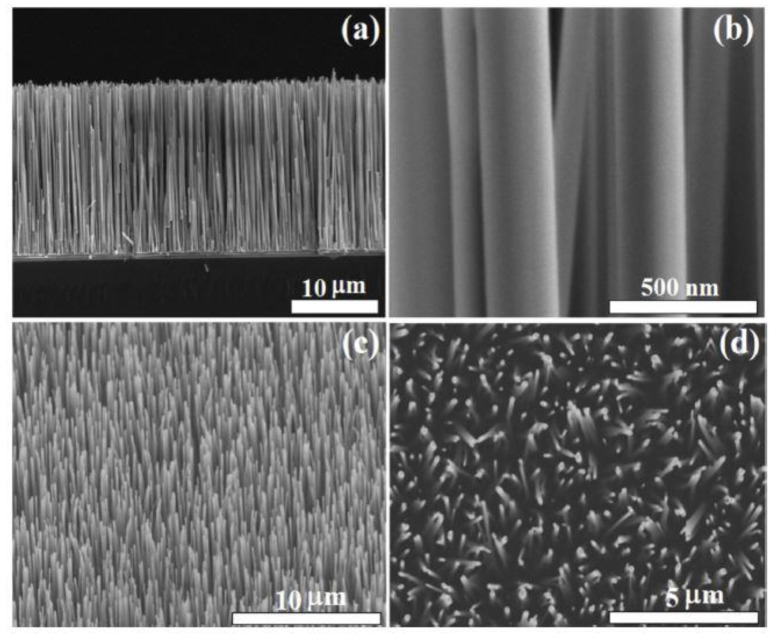
**Field scanning electron microscope** (FESEM), cross-section of ZnO-Nanoroads (NRs) (**a**), closer cross-sectional view of ZnO-NRs (**b**), 25° lateral (**c**) and top view of ZnO-NRs [122] (**d**). Reproduced (2020) with permission from Elsevier.

**Figure 4:**
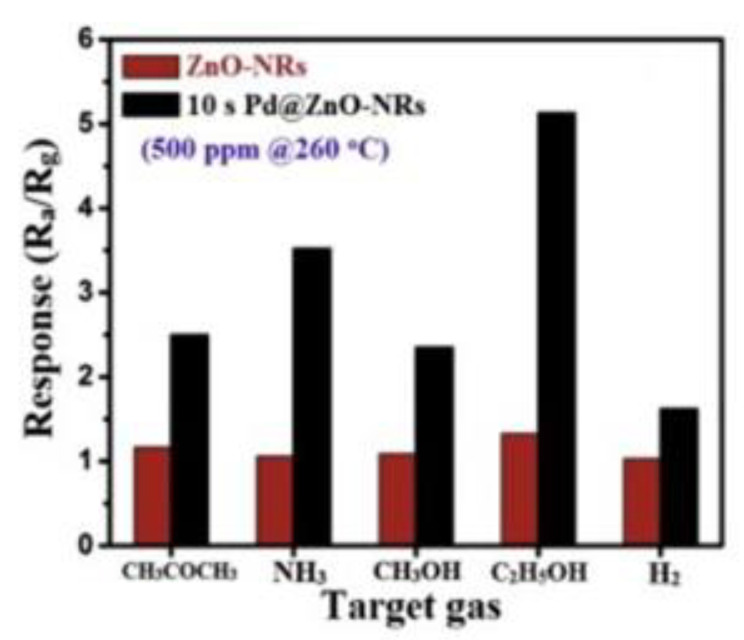
The selectivity of ZnO and Pd@ZnO nanorod sensors at 500 ppm concentrations of different types of gases. [122]. Reproduced (2020) with permission from Elsevier.

1D nanostructures, such as nanorods (Figure 3) [122], are among some of the most used structures in the field of gas sensors due to the advantages related to the large specific surface area, the possibility of adsorption of a large volume of gas molecules, and the ease of their distribution into the volume of the sensitive layer, which leads to a very short response time of the sensor. Cao et al. [122] developed ZnO nanostructures decorated with Pd for ethanol detection, with a chemiresistive gas sensor. ZnO nanorods were grown by chemical vapor deposition, followed by Pd decoration by RF magnetron sputtering. Due to the catalytic activity of Pd and the increased amount of oxygen adsorbed on the surface of the material, the response of the sensor with ZnO nanorods decorated with Pd improved in comparison to the sensor without Pd [122]. The graph in Figure 4 [122] shows both the difference in sensitivity between ZnO and ZnO decorated with Pd for different types of gas, as well as the pronounced selectivity of ZnO decorated with Pd for ethanol.

**Figure 5:**
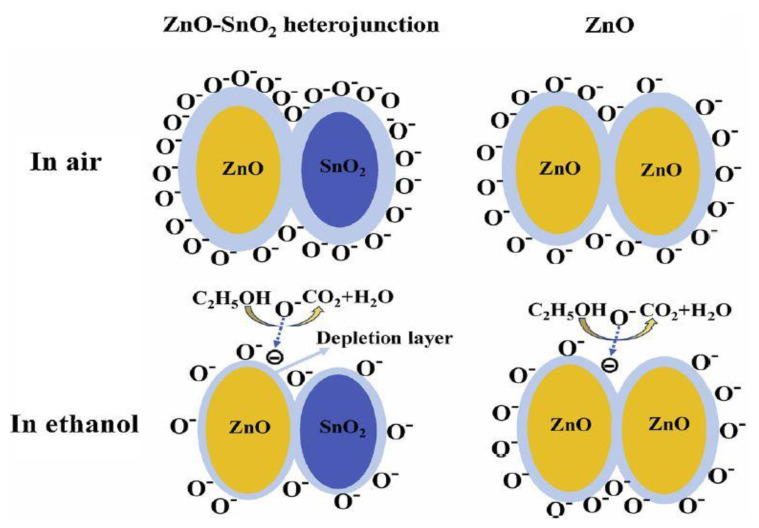
Scheme of the proposed detection mechanism for ZnO and ZnO-SnO_2_ [99]. Reproduced (2020) with permission from Elsevier.

Another method to improve the properties of ZnO is by forming heterostructures with various other oxides, such as ZnO/WO_3_ [144], ZnO/Si [145,146], ZnO/In_2_O_3_ [147] etc. For the detection of 2-methoxyethanol, Shruthu et al. [148] developed two types of sensors: based on Y_2_O_3_ nanoparticles and based on Y_2_O_3_/ZnO nanocomposites. They demonstrated that, based on the n–n heterojunctions, the response of the sensor with Y_2_O_3_/ZnO was greatly improved, obtaining a response time of 17 s and a return time of 21 s. They also found stability in the time and selectivity for 2-methoxyethanol. Qin et al. [99] also demonstrated the improvement of ZnO sensitivity by achieving n–n heterojunctions, developing a mesoporous material of ZnO and SnO_2_. The response of the ZnO/SnO_2_ sensor was 10 times better for ethanol detection, compared to the sensor with ZnO only. Figure 5 [99] illustrates the difference between the interactions of the analyte with the ZnO/SnO2 composite material, compared to that which occurs at the ZnO level. Due to the formation of heterojunctions between ZnO and SnO2, the adsorption of oxygen at the level of ZnO/SnO2 is higher, and the electrostatic interaction with the analyte is more pronounced.

For detection with SAW sensors, Wang et al. [65] developed a ZnO/SiO_2_-based composite for ammonia detection. The best results were obtained for the ZnO: SiO_2_ ratio of 1:2, with a frequency shift of 1.132 kHz for 10 ppm NH_3_, a result much higher than that recorded for the ZnO only sensor. For SAW sensors, the ZnO sensitivity for H_2_S was greatly improved by Tang et al. [82] through using a mesoporous ZnO-Al_2_O_3_ composite. The mesoporous structure of Al_2_O_3_ was inherited by the composite material, which favored the adhesion of H_2_S molecules to the layer. Other results for ZnO-based SAW sensors are presented and analyzed in Table 2 from where, for sensors with composite and nanostructured materials, the sensitivity was higher than for sensors with only ZnO. SAW sensors with ZnO sensitive film demonstrated response and return times of the order of seconds, especially in hydrogen detection. For morphologies that ensure a larger specific area, these times were shorter [149].

To highlight the advantages of using organic materials in combination with inorganic materials, Saaedi et al. [73] highlighted the improvement of the sensor properties with ZnO and PAni-based composites for methanol detection. Thin film deposition was realized under different magnetic flux density by a drop casting method. Following the measurements made for the resistive sensors, researchers found that their operating temperature decreased from 150 °C (the temperature used in the case of ZnO only sensors) to 60 °C (the temperature used by composite ZnO/PAni sensors).

The synthesis method had a direct influence on the obtained morphology. Thus, by methods, such as precipitation [154,155], RF sputtering [156], thermal evaporation [157], electrodeposition [158], electrospinning [117,159], hydrothermal [160,161], micro-wave-assisted solution phase reaction [162], sol gel [163], and pulsed laser deposition [106]), different types of morphologies were obtained, as mentioned above, which are favorable to applications in sensors. 

**Figure 6:**
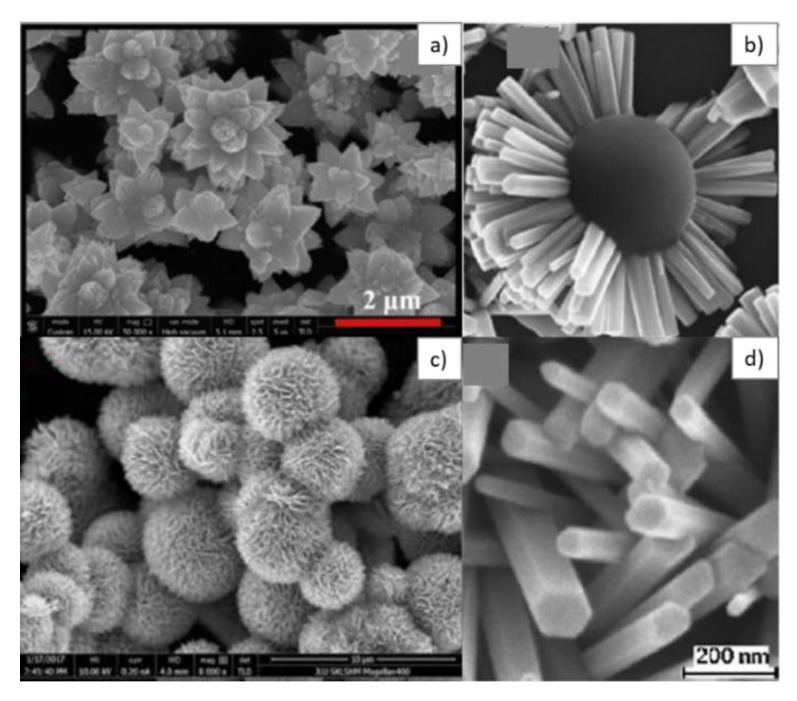
SEM images for ZnO obtained by the hydrothermal method for different morphologies: nanoflowers (**a**,**c**) [84,164], nanowires (**b**,**d**) [91]. Reproduced (2020) with permission from Elsevier.

One of the most widely used synthesis methods for ZnO is the hydrothermal method. This method ensures obtaining well crystalized ZnO, and allows the variation of certain experimental parameters (pressure and pH), so that several types of morphologies can be obtained for the same material using this method [84]. Agarwal et al. [84] synthesized ZnO nanoflowers by the hydrothermal method (Figure 6a), and obtained better sensitivities with resistive sensors to gases, such as ethanol, benzene, carbon monoxide, and nitrogen dioxide, than for other types of morphologies, due to their large specific surface. 

Xie et al. [91] combined pulsed laser deposition with the hydrothermal synthesis method. Thus, they created ZnO seed layers by laser deposition, which were subsequently grown by the hydrothermal method, obtaining ZnO nanowires with good control of the size and morphology (Figure 6b,d). Song et al. [164] highlighted the importance of porosity in the field of sensors, by the synthesis of flower-type nanostructures with and without porosity, using the hydrothermal method. They showed that nanoflower structures (Figure 6c) with porosity resulted in a 1.8-times better response of the resistive sensor, compared with the one without porosity.

The oxygen vacancies from ZnO acted as adsorption sites, electron donor sites, and nucleation centers for small metal clusters [16]. As a result of the ZnO detection mechanism, characterized by the interaction of the analyte with the oxygen adsorbed on the surface of the sensitive material, this material could interact with both reducing and oxidizing gases [152]. This is an electrostatic interaction, which indicates that SAW sensors have the capability of reversibility. In addition, for this type of sensor, the response and recovery times were relatively low, on the order of seconds [152].

Among the gases most targeted for detection using ZnO- and ZnO-based materials in SAW sensors, as shown in Table 2, are hydrogen [45,112], acetone [100,165], hydrogen sulfide [82,166], ethanol [44,100], and ammonia [80,150]. Of these, most RT SAW sensors were made for hydrogen, ammonia and ethanol, for which sensitivities between 11 and 0.005 Hz/ppm were obtained (Table 2) [1].

As demonstrated above, ZnO is a material that achieved remarkable results in the field of sensors. Due to ZnO’s adaptability with different methods of synthesis and processing, its development is easy not only for the field of sensors but for other domains as well. Regarding the ZnO-based sensitive film for SAW sensors, the results were remarkable, particularly for hydrogen, ethanol, and ammonia at RT. Considering the properties of ZnO and how it was developed to meet the performance criteria of SAW sensors, by doping and the synthesis of composite or compound materials, ZnO provides an interesting research area with good perspectives.

## 5. Summary and Outlook

The development of society has led to the increase of the need for control over certain environmental factors, and thus the development of sensors for different gaseous analytes is a permanent concern of researchers.

As discussed above, due to their reliability, sensitivity, short response and return time, small size, etc., SAW sensors are constantly evolving to achieve greater performance in terms of their sensitivity and selectivity. The development of the sensitive layer can be achieved by various unconventional techniques (RF magnetron sputtering, pulsed laser deposition, etc.) through which different types of nanostructures can be obtained, and the sensitivity of the sensor can be considerably increased. Metal oxide semiconductors are among the most utilized materials with reference oxides, such as ZnO, SnO_2_, In_2_O_3_, and SiO_2_. These materials have remarkable sensitivities due to the adsorption capacity of the oxygen on their surface, but also due to the possibility of controlling the morphology to ensure a large specific surface area. RT detection is one of their limitations, but by doping, morphology control, composites or various compounds, this limitation can be overcome. Another limitation of these materials is their selectivity, which can also be improved by use in conjunction with other materials known to have selectivity for a particular analyte.

ZnO is one of the leading representatives of metal oxide semiconductors, known for its good sensitivity to gases, such as hydrogen, ammonia, and other volatile organic compounds. ZnO is a versatile material, and can be synthesized by a variety of methods, most commonly, the hydrothermal method, through which morphologies, such as nanoflowers and nanorods, which are favorable for sensors, can be obtained. ZnO also allows doping and the development of composites and compounds, thus favoring the development of high-performance materials. Using ZnO-based sensitive materials, SAW sensors led to remarkable results, which were obtained at RT.

The advantages of ZnO as a sensitive material, including being relatively easy to develop and cheap, with good sensitivity, encourage the study of its development, particularly in the direction of obtaining different ZnO-based compounds, under different morphologies, to obtain greater sensitivity, selectivity, and produce response and return times that are as small as possible. Considering the variety of metals with which ZnO can be doped, oxides with different heterojunctions can be obtained, and ZnO remains one of the materials of interest for the study of sensors. The disadvantage of the lack of sensitivity at room temperature, as observed, can be overcome by developing nanostructures, composite materials, and compounds of ZnO.

Regarding the use of ZnO for the development of SAW sensors, ZnO can be adapted for deposition by a series of methods, and the properties presented above led to great advantages in the form of excellent sensitivities and selectivities.

## Figures and Tables

**Table 1:** Types of ZnO based sensors.

Nr. crt	Sensor Type	Sensitive Material	Analyte	Minimum ConcentratIon Detected	Operating Condition	References
1	Resistive	ZnO nanoflowers	Ethanol	5 ppm	250 °C	[84]
			Nitrogen dioxide	250 ppb	200 °C	
			Benzene	2.5 ppm	250 °C	
2	Resistive	ZnO microspheres	Acetone	100 ppm	280 °C	[129]
3	Optoelectronic	ZnO/Pd	Nitrogen dioxide	2.5 ppb	RT	[71]
4	Chemiresistive	Pd/ZnO	Ethanol	500 ppm	260 °C	[122]
5	Resistive	C/ZnO/ZnO	Ethanol	100 ppm	300 °C	[90]
6	Resistive	Au doped ZnO	Acetone	5 ppb	150 °C	[130]
		Pd doped ZnO				
7	Chemiresistive	ZnO nanowire-integrated film	Nitrogen dioxide	50 ppb	RT, UV activation	[11]
8	Resistive	ZnO microspheres	Hydrogen	100 ppm	225 °C	[131]
9	Chemiresistive	Polyvinyl pyrrolidine-ZnO nanofibers	Ammonia	20 ppm	RT	[124]
10	SAW	ZnO-Al2O3 composite	Hydrogen sulphide	500 ppb	RT	[81]

**Table 2:** Results of SAW sensors based on ZnO for different types of gases.

Nr. Crt.	Sensitive Material	Analyte	Sensitivity	Operating Condition	Response/Recovery Time	References
1	ZnO	Hydrogen	0.15 Hz/ppm	RT	12–16 s/-	[45]
	Pd/ZnO		0.51 Hz/ppm			
2	ZnO-Al2O3 composite	Hydrogen sulfide	15 kHz/ppm	RT	-	[82]
3	ZnO nanowires	Hydrogen	0.062 Hz/ppm	RT	-	[112]
	ZnO thin film		0.010 Hz/ppm		-	
4	ZnO nanofilm	Ammonia	3 Hz/ppm	RT	50 s/34 s	[150]
	ZnO nanorods		11 Hz/ppm		226 s/431 s	
5	ZnO/CuO	2-propanol	200.26 kHz/100 ppm	RT	-	[100]
		Acetone	107.23 kHz/100 ppm		-	
		Ethanol	100.69 kHz/100 ppm		-	
6	ZnXFeyO	Oxygen	-258.85 Hz/1%O2	RT	200 s/-	[151]
7	ZnO nanowire (600 nm)	Hydrogen	0.015 Hz/ppm	RT	9–15 s/6–9 s	[149]
		Deuterium	0.09 Hz/ppm		9–15 s/6–9 s	
	ZnO Film (100 nm)	Hydrogen	0.005 Hz/ppm		11–18 s/7–11 s	
		Deuterium	0.026 Hz/ppm		11–18 s/7–11 s	
8	ZnO/SiO2	Ammonia	0.1132 kHz/ppm	-	-	[80]
9	Multi-crystal ZnO	UV	15.790 kHz	-		[152]
	ZnO nanowire		101.340 kHz		-	
10	ZnO nanoparticle film	Hydrogen	55 kHz/1% H2	RT	-	[153]
